# Limb ischemic preconditioning ameliorates renal microcirculation through activation of PI3K/Akt/eNOS signaling pathway after acute kidney injury

**DOI:** 10.1186/s40001-020-00407-4

**Published:** 2020-03-19

**Authors:** Cheng Chen, Li Sun, Wanfen Zhang, Yushang Tang, Xiaoping Li, Ran Jing, Tongqiang Liu

**Affiliations:** 1 Division of Nephrology, The Affiliated Changzhou No. 2 People’s Hospital of Nanjing Medical University, Changzhou, 213003 Jiangsu China; 2 Division of Nephrology, Xuyi People’s Hospital, Huaian, 211700 Jiangsu China

**Keywords:** Acute kidney injury, Limb ischemic preconditioning, PI3K, Akt/eNOS, Wortmannin

## Abstract

**Purpose:**

Contrast-induced acute kidney injury (CI-AKI) resulting from administration of iodinated contrast media (CM) is the third leading cause of hospital-acquired acute kidney injury and is associated with substantial morbidity and mortality. Deteriorated renal microcirculation plays an important role in CI-AKI. Limb ischemic preconditioning (LIPC), where brief and non-injurious ischemia/reperfusion is applied to a limb prior to the administration of the contrast agent, is emerging as a promising strategy for CI-AKI prevention. However, it is not known whether the renal protection of LIPC against CI-AKI is mediated by regulation of renal microcirculation and the molecular mechanisms remain largely unknown.

**Methods:**

In this study, we examined the renal cortical and medullary blood flow in a stable CI-AKI model using 5/6-nephrectomized (NE) rat. The LIPC and sham procedures were performed prior to the injection of CM. Furthermore, we analyzed renal medulla hypoxia using in vivo labeling of hypoxyprobe. Pharmacological inhibitions and western blotting were used to determine the underlying molecular mechanisms.

**Results:**

In this study, we found LIPC significantly ameliorated CM-induced reduction of medullary blood flow and attenuated CM-induced hypoxia. PI3K inhibitor (wortmannin) treatment blocked the regulation of medullary blood flow and the attenuation of hypoxia of LIPC. Phosphorylation of Akt/eNOS was significantly decreased via wortmannin treatment compared with LIPC. Nitric oxide synthase-inhibitor [Nω-nitro-l-arginine methyl ester (L-NAME)] treatment abolished the above effects and decreased phosphorylation of eNOS, but not Akt.

**Conclusions:**

Collectively, the results demonstrate that LIPC ameliorates CM-induced renal vasocontraction and is mediated by activation of PI3K/Akt/eNOS signaling pathway.

## Background

With the increasing use of iodinated contrast medium (CM) in diagnostic and interventional procedures, CM-induced acute kidney injury (CI-AKI) has become the third most common cause for hospital-acquired AKI [1]. The development of CI-AKI is associated with increased morbidity, prolonged hospitalization, cardiovascular events, persistent kidney damage and higher mortality, in addition to increased caregiver burden and higher financial cost [2]. The prophylactic strategies developed to mitigate CI-AKI are largely restricted to pre- and post-hydration protocols and attempts of pharmacological interventions have been disappointing [3].

The pathophysiology of CI-AKI is complex and far from being elucidated; however, two major pivotal factors are recognized: (1) vasoconstriction and renal ischemic injury; (2) renal tubular cytotoxicity [4, 5]. CM administration induces transient and intense vasoconstriction, resulting from release of vasoconstrictors such as adenosine and endothelin, as well as inhibition of vasodilators such as nitric oxide (NO) and prostaglandins, causing medullary ischemia and hypoxia [4, 6]. Furthermore, iodinated contrast agent and oxygen-free radical have direct cytotoxic effects, which mediate direct tubular injury, inducing vacuolization, change in mitochondrial function, and even apoptosis [7].

Limb ischemic preconditioning (LIPC) is a strategy of applying transient, brief episodes of ischemia to induce resistance in a target organ against the oxidative stress and injury caused by a larger ischemic insult [8], and may offer a highly appealing, non-invasive, nonpharmacological and cost-effective strategy on CI-AKI prevention. Indeed, growing number of clinical reports have shown the beneficial effects of LIPC against CI-AKI [9–11]. A recent meta-analysis concluded demonstrated that LIPC could effectively exert reno-protective role and significantly decrease the incidence of CI-AKI [12].

Despite growing understanding of the mechanisms of renoprotection by LIPC, it is not clear whether LIPC elicits ameliorative effect on renal microcirculation in CI-AKI. Accumulating evidence demonstrates that the important role of nitric oxide/nitrite in LIPC and/or CI-AKI [7, 13, 14], and “reperfusion injury salvage kinase” (RISK) pathway has been found to be an important target of LIPC-induced protection against CM-AKI, in which Phosphatidylinositol3-kinase (PI3K)/Akt signaling pathway is involved in mediating LIPC-induced renoprotection [15, 16]. Endothelial nitric oxide synthase (eNOS) is a key enzyme in the regulation of endothelial-derived NO production [17]. Akt has also been linked to renal NO production through activation of eNOS [18].

Taken together, we hypothesized that LIPC ameliorated renal microcirculation through activation of PI3K/Akt/eNOS signaling pathway. In this study, we investigated the renal cortical and medullary blood flow in a stable CI-AKI model using 5/6-nephrectomized (NE) rat and examined the underlying molecular signaling pathway using pharmacological inhibitors.

## Materials and methods

### Chemicals and reagents

The nonionic CM used was low-osmolar iohexol (350 mg iodine/mL, 844 mOsm/kg of water and 10.4 cPs at 37 °C, GE Healthcare, Shanghai, China). Wortmannin and Nω-nitro-l-arginine methyl ester (L-NAME) were obtained from Abcam (Cambridge, MA, USA), which were dissolved in dimethyl sulfoxide (DMSO) (Sigma Inc, St. Louis, MO, USA) and diluted in saline so that the vehicle constituted less than 1% of the total volume injected.

### Animals and establishment of CI-AKI model

Male Sprague–Dawley rats (180–200 g) were obtained from the Animal Center of The Affiliated Changzhou No. 2 People’s Hospital of Nanjing Medical University, China. The rats were acclimatized for 7 days before the start of study and handled in accordance with the institutional and national guidelines for animal research. The 5/6 nephrectomy (NE) procedure was performed on animals anesthetized with intraperitoneal injection of 4% sodium pentobarbital (40 mg/kg). The procedure involved NE of the right kidney and resection of two-thirds of the left kidney, as described previously [16, 19]. The CI-AKI model used 5/6 NE rats 6 weeks after the NE surgery and was established by dehydration for 48 h, followed by administration of 10 mL/kg body weight (3.5 gI/kg) iohexol via the tail vein. All animals had ad libitum access to water and food after the injection.

### Grouping

Animals of CI-AKI model was randomly divided into four groups as follows:

1. CM + Sham
2. CM + LIPC
3. CM + LIPC + WORT
4. CM + LIPC + L-NAME

### Measurement of renal microcirculation

Animals of CI-AKI model were anesthetized with 2% isoflurane vaporized by oxygen, laced on a small-animal operating table. The left kidney was exposed through a midline incision, decapsulated, and mechanically fixed. The body and renal temperatures were monitored and maintained at approximately 37 °C with a heating lamp and intermittent dripping of warm saline and paraffin oil [20]. A Dual-Channel Laser Doppler flowmeter, PeriFlux 5000, (Perimed, Sweden) was used to measure renal microcirculation. Laser Doppler fibers were inserted into the renal cortex to a depth of 2 mm and into the renal medulla to a depth of 4 mm. Measurements were expressed as arbitrary perfusion units that represent the product of the velocity and the concentration of moving blood cells within the measuring volume. After 5 min of equilibration, basal readings were recorded and subsequent readings were recorded every 5 min [13, 21].

### Limb ischemic preconditioning

An incision was made on the femoral triangle under a moderate level of ether anesthesia and local anesthesia with a 1% procaine solution. The left lateral femoral arteries of the rats were dissected out and clamped four times for 5 min each, separated by 5-min intervals. The wound was sutured after the LIPC. The sham operation for LIPC included all surgical procedures or treatments except the clamping of the femoral arteries.

### In vivo labeling of hypoxia

Renal tissue hypoxia was assessed at 15 min or 30 min after an intravenous injection of saline or CM using Hypoxyprobe-1 Omni Kit (Natural Pharmacia International Inc., Burlington, MA, USA), which contains pimonidazole hydrochloride and rabbit polyclonal anti-pimonidazole. Pimonidazole HCl remains in the hypoxic cells upon forming an irreversible adduct with thiol groups in tissues with a PO_2_ < 10 mmHg [22].

The protein adducts are effective immunogens for rabbit anti-pimonidazole antisera. At 60 min prior to being killed, each rat received injections via the tail vein of hypoxyprobe-1 in a 0.5-mL bolus (60 mg/kg bw). Immunohistochemical staining procedure was identical with immunohistochemical staining for pimonidazole, except the primary antibody used was rabbit polyclonal anti-pimonidazole (1:500) for 40-min incubation at room temperature. The specimens were scored by the extent and intensity of hypoxyprobe.

### Immunohistochemical staining for phosphorylated-eNOS

Immunohistochemical staining was performed on 3-µm paraffinized sections. The samples were dewaxed and dehydrated, washed in phosphate-buffered saline (PBS), and incubated with 3% H_2_O_2_ for 10 min to eliminate endogenous peroxidase activity and then treated with normal goat serum (1:20) for 20 min. Next, the samples were incubated with anti-p-eNOS antibody (rabbit polyclonal, 1:200; Santa Cruz) at 4 °C overnight. The sections were then incubated with a horseradish peroxidase-conjugated secondary antibody (anti-rabbit IgG). After rinsing three times with PBS, the sections were stained with 3,3′-diaminobenzidine (Sigma, Shanghai, China), counterstained with hematoxylin, and then evaluated under a light microscope. The stained specimens were assessed by a pathologist in a blinded fashion. We randomly selected five high-magnification (200×) fields of the renal medulla. The specimens were scored according to the percentage of p-eNOS positive cells and the extent and the intensity of staining.

### Western blot assay

Equal amounts of proteins (40 μg total protein or 80 μg nuclear protein per lane) were loaded onto a 12.5% gradient 2-amino-2-(hydroxymethyl)propane-1,3-diol hydrochloride (Tris–HCl) sodium dodecyl sulfate–polyacrylamide gel and then transferred to a polyvinylidene difluoride membrane. Nonspecific binding to the membrane was blocked for 1 h at room temperature with 5% nonfat milk in 1 × TBS, followed by incubation with primary antibodies against total Akt (rabbit monoclonal 1 :1000; Cell Signaling Technology, Danvers, MA), p-Akt (Ser473, rabbit monoclonal 1:2,000; Cell Signaling Technology), total eNOS (rabbit monoclonal 1:1000; 1:250 dilution, Cell Signaling, USA), and p-eNOS (Ser1179, 1:250 dilution, Invitrogen, USA) overnight at 4 °C. After washing with TBST three times, membranes were incubated with horseradish peroxidase-conjugated rabbit or goat secondary antibody (1:10,000 dilution; Kang Chen Biotechnology, Guangzhou, China) for 1 h at room temperature, followed by three washes for 10 min each. Blots were developed using enhanced chemiluminescent reagents (Thermo Fisher Scientific, Pittsburgh, PA, USA) and target band density was scanned using an LAS-3000 detection system. Image J software was used to analyze band intensities.

### Statistical analysis

Data are presented as mean ± standard deviation (SD). Statistical differences between conditions were determined with GraphPad Prism software. Data were analyzed by one-way analysis of variance (ANOVA) with Tukey's multiple comparisons (parametric tests) or Kruskal–Wallis test with Dunns’ multiple comparisons (nonparametric tests). Statistical significance of difference was defined as a *P* value < 0.05.

## Results

### CM administration altered renal microcirculation and induced hypoxia

**Fig. 1:**
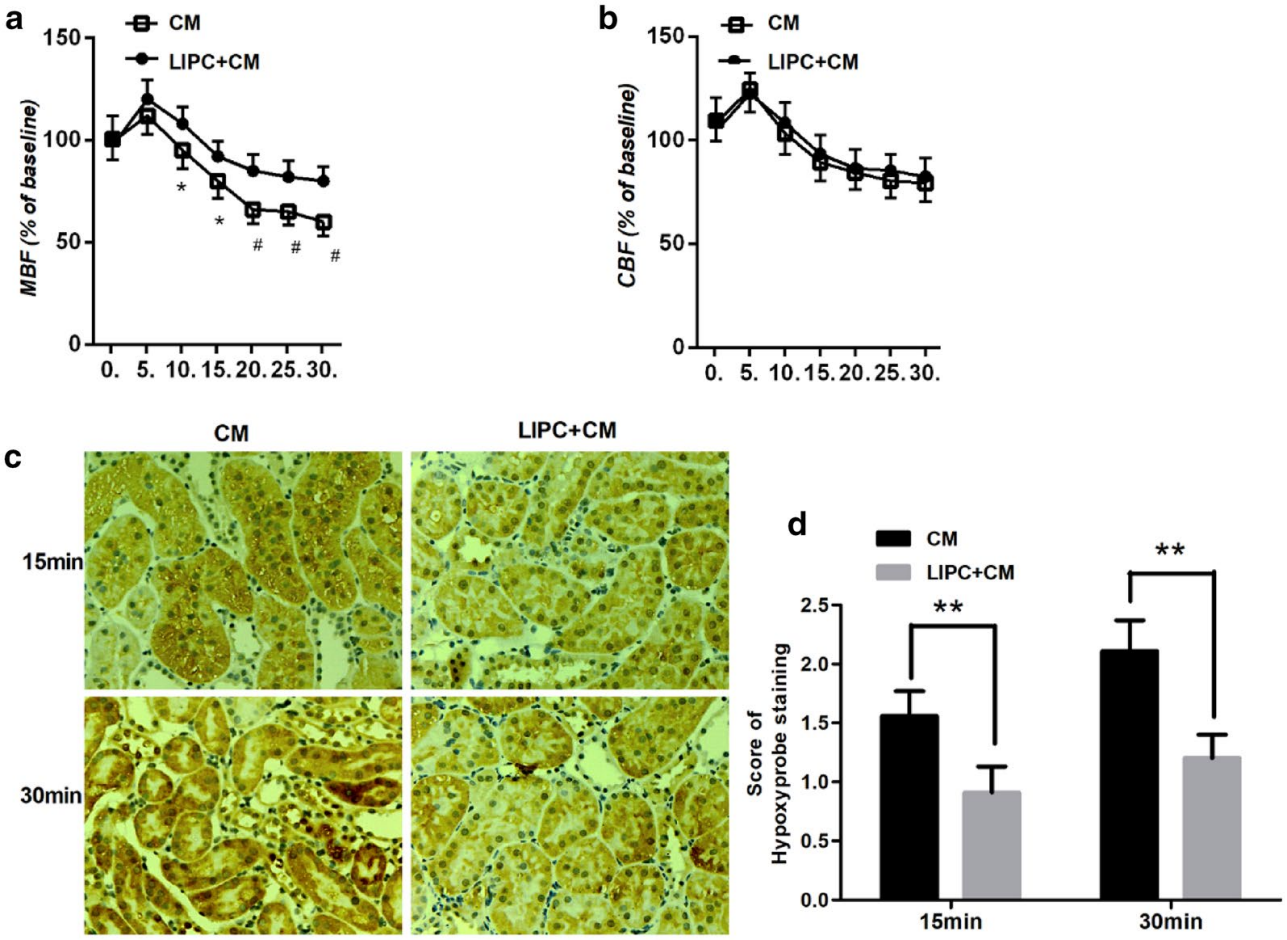
CM administration-induced vasoconstriction was attenuated significantly following LIPC in the medulla (**a**) but not in the cortex (**b**). Representative photomicrographs of kidney sections of CI-AKI with hypoxyprobe staining (**c**). LIPC significantly decreased score of hypoxyprobe staining in 15 and 30 min followed by CM administration (**d**). Original magnification ×200. *P < 0.05, **P < 0.01, ^#^*P* < 0.01; *n* = 7 each. The values shown are the mean ± SD

To examine renal microcirculation change in our CM-AKI model and the effect of LIPC, we used a dual-channel Laser Doppler flowmeter to determine renal cortical and medullary blood flow. CM administration induced a transient decrease of both renal medullary and cortical blood flow. Compared with CM + Sham group, we found that LIPC significantly ameliorated CM-induced reduction of medullary blood flow, but not cortical blood flow, 10 min after CM administration (Fig. 1a, b).

We then examine the effect of LIPC on hypoxia condition of CM-AKI. We used in vivo labeling of hypoxyprobe to determine tissue hypoxia 15 min and 30 min followed by CM administration. We found that LIPC significantly alleviated hypoxia condition of renal tissue after 15 and 30 min of CM administration (Fig. 1c, d), suggesting the important role of microcirculation in LIPC against CI-AKI.

### Inhibition of PI3K abolished the protective effects on renal microcirculation and hypoxia of LIPC against CI-AKI

**Fig. 2:**
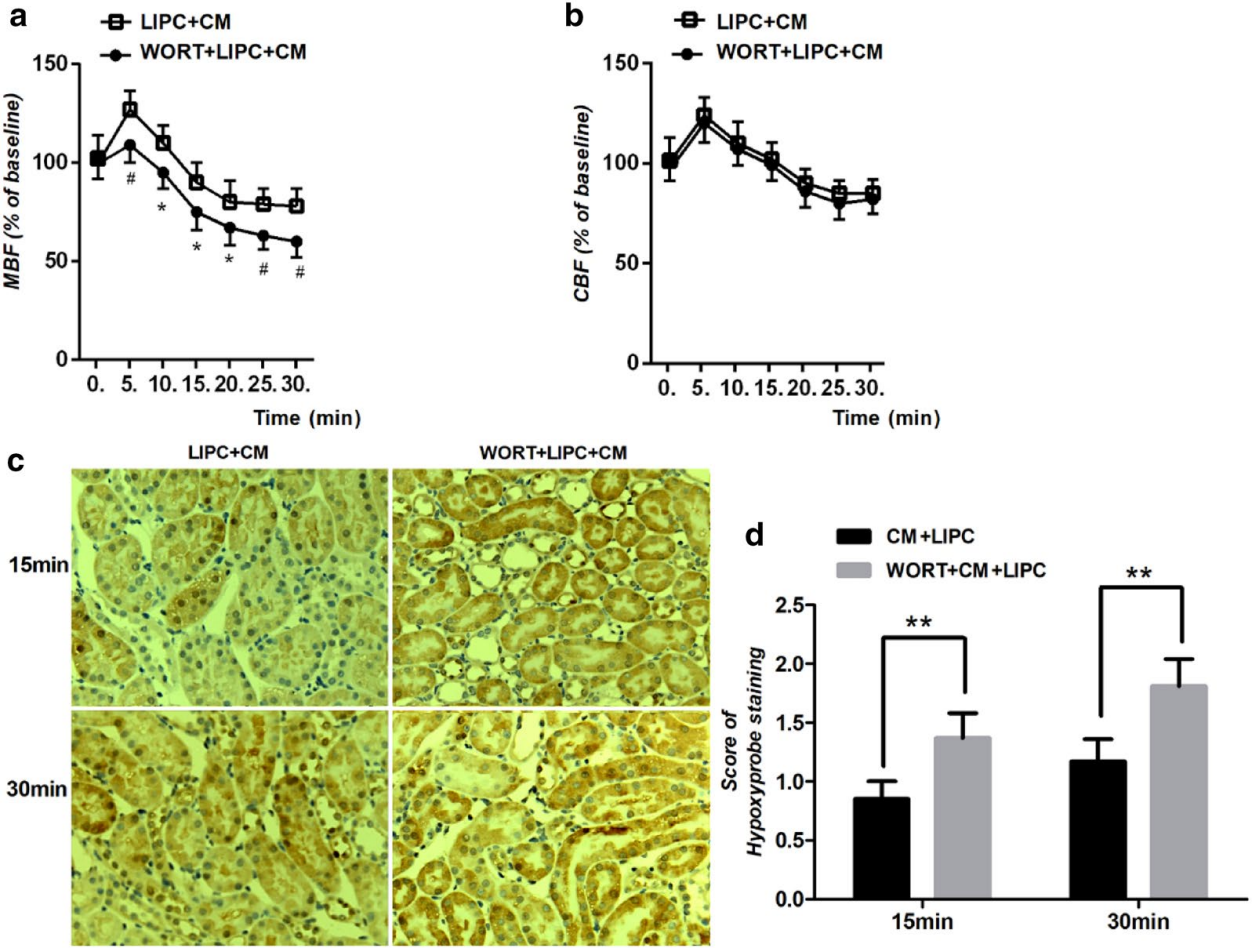
Inhibition of PI3K abolished the renoprotective effects of LIPC on microcirculation and hypoxia following in CM-AKI. WORT (wortmannin) significantly decreased renal medullary blood flow (**a**), but not cortical blood flow (**b**) in LIPC + CM. Representative photomicrographs of kidney sections of CI-AKI with Hypoxyprobe staining (**c**). WORT significantly increased score of Hypoxyprobe staining in 15 and 30 min in CM + LIPC (**d**). Original magnification × 200. *P < 0.05, **P < 0.01, ^#^*P* < 0.01; *n* = 6 each. The values shown are the mean ± SD

To explore the role of reperfusion injury salvage kinases (RISK) pathway in LIPC against CI-AKI, we first pharmacologically inhibited PI3K using PI3K inhibitor (wortmannin). We examined renal microcirculation and tissue hypoxia and found that inhibition of PI3K abolished the regulation of medullary blood flow and the attenuation of hypoxia of LIPC. Renal medullary blood flow was significantly decreased compared with LIPC + CM group, but not cortical blood (Fig. 2a, b). Scores of hypoxyprobe staining were significantly increased compared with LIPC + CM group (Fig. 2c, d).

### Inhibition of PI3K reduced the activation of Akt/eNOS pathway

**Fig. 3:**
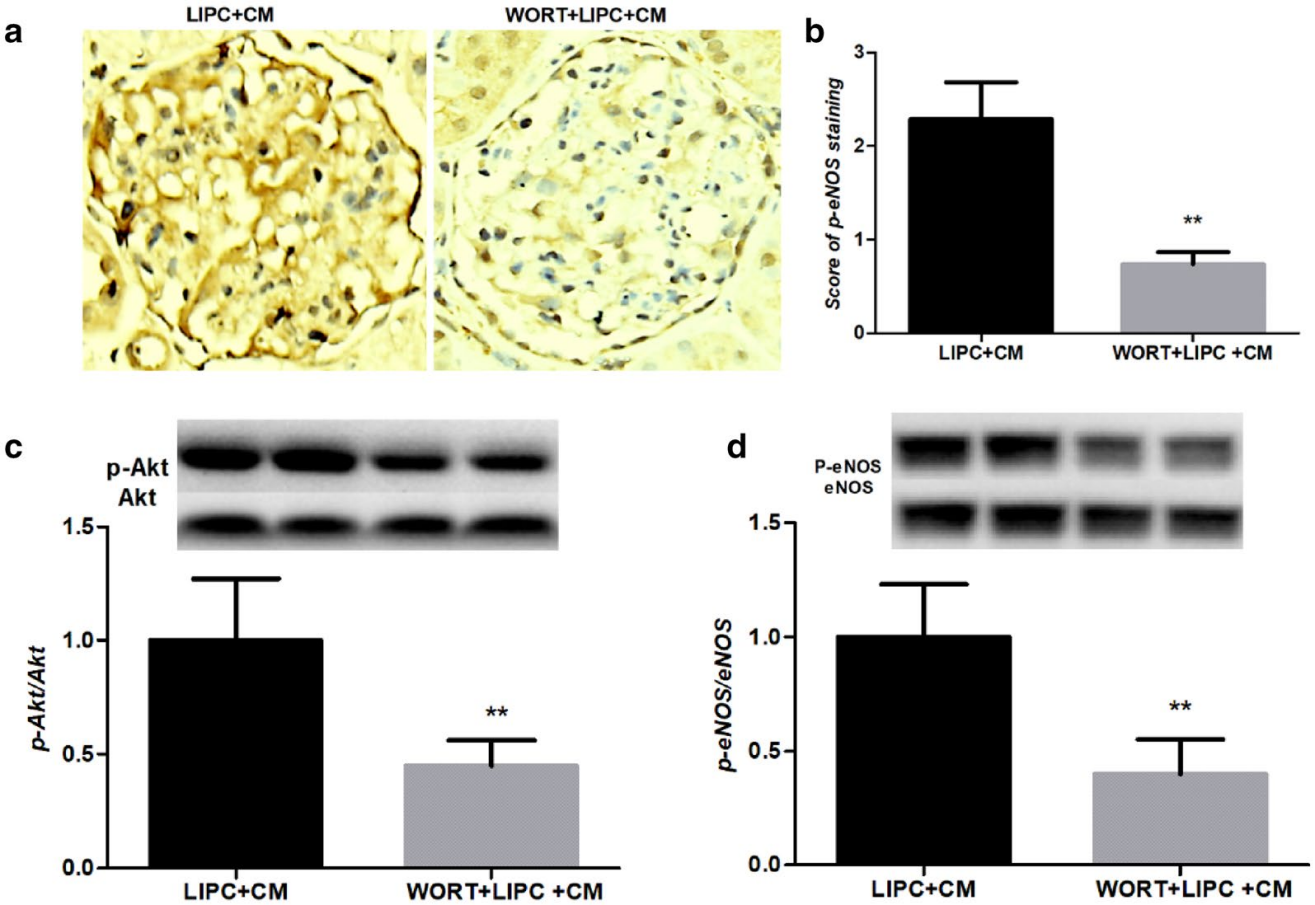
Inhibition of PI3K significantly decreased phosphorylation of Akt and eNOS. Representative photomicrographs of kidney sections of CI-AKI sections with p-eNOS staining (**a**). Score of p-eNOS staining was significantly decreased in WORT + LIPC + CM (**b**). Representative western blots and quantitative analysis of p-Akt and Akt (**c**), p-eNOS and eNOS (**d**). WORT significantly decreased the ratios of phosphorylated Akt and eNOS in LIPC + CM. Original magnification ×400. **P < 0.01; *n* = 6 each. The values shown are the mean ± SD

To further explore the role of RISK pathway in LIPC against CI-AKI, we immunohistochemical stained p-eNOS in kidney sections. We found that the score of p-eNOS staining was significantly reduced via PI3K inhibition (Fig. 3a, b). We further examined the protein phosphorylation level using Western blotting analysis. The ratios of phosphorylated Akt and eNOS were significantly decreased compared with LIPC + CM (Fig. 3c, d), suggesting that the ameliorated renal microcirculation and hypoxia is mediated by PI3K/Akt/eNOS signaling pathway in LIPC against CI-AKI.

### Inhibition of eNOS abolished the protective effects on renal microcirculation and hypoxia of LIPC against CI-AKI

**Fig. 4:**
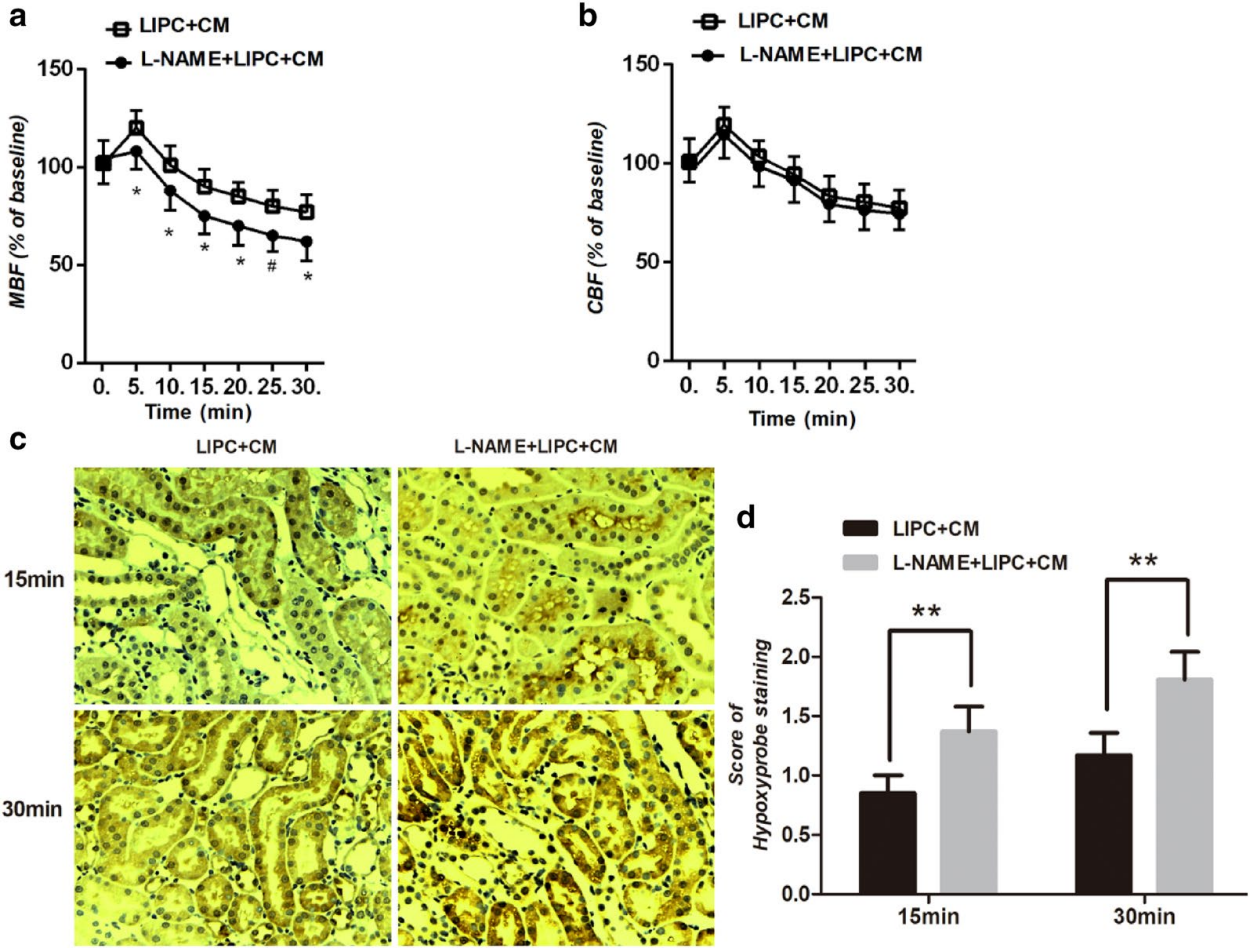
Inhibition of eNOS abolished the regulation of microcirculation and hypoxia of LIPC against CI-AKI. L-NAME significantly decreased renal medullary blood flow (**a**), but not cortical blood flow (**b**) in LIPC + CM. Representative photomicrographs of kidney sections of CI-AKI with Hypoxyprobe staining (**c**). L-NAME significantly increased score of Hypoxyprobe staining in 15 and 30 min in CM + LIPC (**d**). Original magnification ×200. *P < 0.05, **P < 0.01, ^#^*P* < 0.01; *n* = 6 each. The values shown are the mean ± SD

To further examine the role of eNOS in LIPC against CI-AKI, we pharmacologically inhibited eNOS using L-NAME and examined renal microcirculation and tissue hypoxia. Renal medullary blood flow was significantly decreased compared with LIPC + CM group, but not cortical blood (Fig. 4a, b). Scores of hypoxyprobe staining were significantly increased compared with LIPC + CM group (Fig. 4c, d).

### L-NAME reduced the phosphorylation of eNOS, but not Akt

**Fig. 5:**
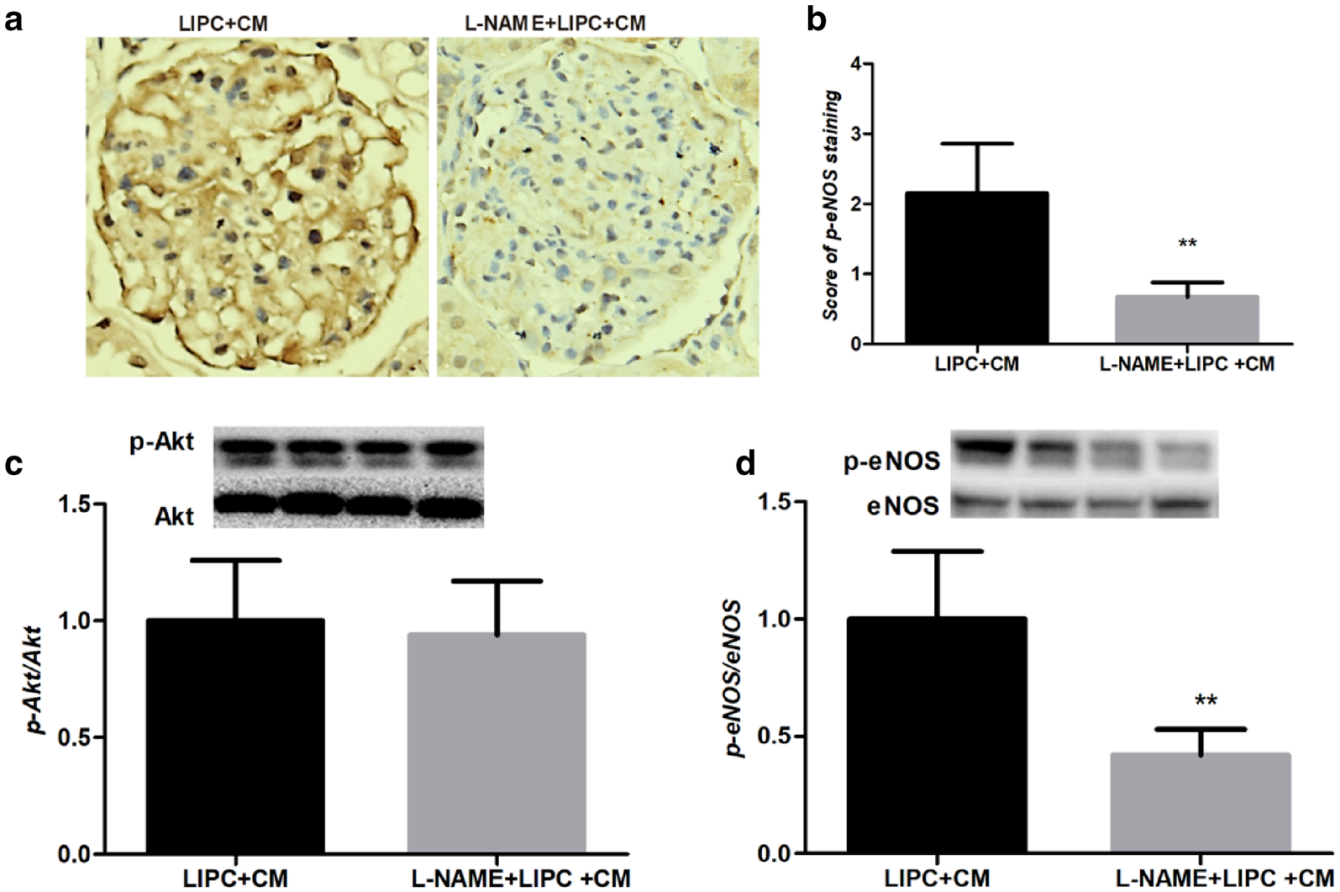
Inhibition of NOS significantly decreased phosphorylation of Akt and eNOS. Representative photomicrographs of kidney sections of CI-AKI sections with p-eNOS staining (**a**). Score of p-eNOS staining was significantly decreased in L-NAME + LIPC + CM (**b**). Representative western blots and quantitative analysis of p-Akt and Akt (**c**), p-eNOS and eNOS (**d**). L-NAME significantly decreased the ratios of phosphorylated eNOS, but not Akt in LIPC + CM. Original magnification ×400. **P < 0.01; *n* = 6 each. The values shown are the mean ± SD

We then examine the role of eNOS in RISK pathway of LIPC against CI-AKI. We used immunohistochemical staining and Western blotting analysis to determine the phosphorylation of eNOS and Akt. Nitric oxide synthase inhibitor, L-NAME, significantly decreased the phosphorylation level of eNOS (Fig. 5a, b, d), but not Akt (Fig. 5c), suggesting the essential role of eNOS in PI3K/Akt/eNOS signaling pathway in LIPC against CI-AKI.

## Discussion

The alteration of renal microcirculation due to CM administration is considered a key pathophysiology mechanism of CM-AKI [3]. CM induced a transient and intense renal vasocontraction especially in medulla [23, 24]. Medulla receives only ~ 10% of total renal prefusion even in functionally normal kidney [25], making it a more severe hypoxia environment [26]. Animal studies indicate that all CM, irrespective of osmolality, reduce velocity and increase aggregation of red blood cells in the renal medullary vessels [24, 27]. Previous study also reported reduced medullary blood flow but increased cortical flow following administration of both high- and low-osmolality CM in rats [23]. Our results demonstrate that LIPC ameliorates the vasoconstriction following CM administration in the renal medulla, but not in the cortex, supporting that reduced medullary flow plays an important role in the pathogenesis of CM-AKI.

It has not been fully examined whether LIPC exert beneficial effects on renal microcirculation. Studies using 3T functional magnetic resonance imaging (fMRI) suggested that on humans, LIPC alone could induce a higher oxygen content in kidney tissue [28], as well as increasing intra-renal perfusion [29]. Studies using laser Doppler flowmetry reported improved microcirculation on other organs, such as liver in Ischemia/Reperfusion (IR) model [30, 31], and spinal cord blood flow [32]. In the present study, we first reported the amelioration of impaired renal medulla blood flow of LIPC in CI-AKI, and alleviated hypoxia condition of renal tissue after 15 and 30 min of CM administration, suggesting the important role of microcirculation in LIPC against CI-AKI.

Various studies have demonstrated the relationship between nitrite/nitric oxide (NO) and CM-induced vasoconstriction, as NO synthesis is down-regulated by CM [21], and the decreased renal blood flow in CI-AKI can be restored by l-arginine (a NO precursor) [33] and further decreases by L-NAME [13]. Studies suggest that the generation of NO and the activation of eNOS play important roles in the protective effects of LIPC [34, 35]. The reperfusion injury salvage kinases (RISK) pathway that activated in LIPC, such as the phosphatidylinositol-3 kinase/Akt (PI3K/Akt) pathway which involved in signal transduction related to cell growth, proliferation, differentiation, motility, survival and metabolism, plays a key part in LIPC [36, 37]. Akt, which is activated by phosphorylation via activated PI3K, phosphorylates eNOS on serine 1177 (p-eNOS), thereby activating this enzyme [38, 39]. The protective effect of this signaling pathway, PI3K/Akt/eNOS, has been confirmed in many studies [15, 40], including LIPC [41, 42]. In this study, we validated the activation of this signaling pathway, and also demonstrated its role in regulating renal microcirculation. Pharmacological inhibition of PI3K or eNOS abrogated the regulation of renal microcirculation in LIPC, and restored the hypoxia condition induced by CM. However, it is important to note that the recruitment of pro-survival kinases is protective when acutely activated, while their chronic activation would be considered to be harmful and proinflammatory [43, 44]. Second, the pathway must be activated before insult like reperfusion or chemical agent to be nonprotective [37].

Even though, it is important to note that the protective effect of LIPC is not only due to ameliorated microcirculation mediated by NO. Study showed that the decrease in NO is related to increased local production of reactive oxygen species (ROS) [45], with NO being an antioxidant, NO may exert its protective effect via reducing oxidative stress [46]. Moreover, study has showed that CM-induced direct cell damage leads to oxidative stress and low-nitric oxide levels in medullary thick ascending limbs of the Henle's loop in an environment where tissue hypoxia is absent [14]. It has been shown that NO also directly affects the oxygen availability for regulating the mitochondrial oxygen utilization [47–49].

eNOS/NO is important for promoting endothelial cells proliferation, adhesion, migration and angiogenesis of progenitor cells [50]. Study showed that LIPC induced great accumulation of endothelial progenitor cells (EPCS) in renal medulla, suggesting that the renal protection of LIPC partly exerts on EPCs in promoting proliferation and angiogenesis [51]. Thus, it would be very interesting to examine the eNOS activity on recruited EPCs followed by LIPC in CI-AKI.

## Conclusions

Our study demonstrates that the LIPC ameliorates CM-induced renal vasocontraction and attenuates CM-induced hypoxia. The regulation of microcirculation is mediated by activation of PI3K/Akt/eNOS signaling pathway.

## Data Availability

The data used to support the findings of this study are available from the corresponds author upon request.

## References

[1] Go RS, Adjei AA (1999). Review of the comparative pharmacology and clinical activity of cisplatin and carboplatin. J Clin Oncol.

[2] Mitchell AM, Jones AE, Tumlin JA (2010). Incidence of contrast-induced nephropathy after contrast-enhanced computed tomography in the outpatient setting. Clin J Am Soc Nephrol.

[3] Dugbartey GJ, Redington AN (2018). Prevention of contrast-induced nephropathy by limb ischemic preconditioning: underlying mechanisms and clinical effects. Am J Physiol Renal Physiol.

[4] Pistolesi V, Regolisti G, Morabito S (2018). Contrast medium induced acute kidney injury: a narrative review. J Nephrol.

[5] Atanda AC, Olafiranye O (2017). Contrast-induced acute kidney injury in interventional cardiology: emerging evidence and unifying mechanisms of protection by remote ischemic conditioning. Cardiovasc Revasc Med.

[6] Heyman SN, Rosen S, Rosenberger C (2008). Renal parenchymal hypoxia, hypoxia adaptation, and the pathogenesis of radiocontrast nephropathy. Clin J Am Soc Nephrol.

[7] Golshahi J, Nasri H, Gharipour M (2014). Contrast-induced nephropathy; a literature review. J Nephropathol.

[8] Murry CE, Jennings RB, Reimer KA (1986). Preconditioning with ischemia: a delay of lethal cell injury in ischemic myocardium. Circulation.

[9] Menting TP, Sterenborg TB, de Waal Y (2015). Remote ischemic preconditioning to reduce contrast-induced nephropathy: a randomized controlled trial. Eur J Vasc Endovasc Surg.

[10] Er F, Nia AM, Dopp H (2012). Ischemic preconditioning for prevention of contrast medium-induced nephropathy: randomized pilot RenPro Trial (Renal Protection Trial). Circulation.

[11] Koch C, Chaudru S, Lederlin M (2016). Remote ischemic preconditioning and contrast-induced nephropathy: a systematic review. Ann Vasc Surg.

[12] Zhou CC, Yao WT, Ge YZ (2017). Remote ischemic conditioning for the prevention of contrast-induced acute kidney injury in patients undergoing intravascular contrast administration: a meta-analysis and trial sequential analysis of 16 randomized controlled trials. Oncotarget.

[13] Myers SI, Wang L, Liu F (2006). Iodinated contrast induced renal vasoconstriction is due in part to the downregulation of renal cortical and medullary nitric oxide synthesis. J Vasc Surg.

[14] Liu ZZ, Schmerbach K, Lu Y (2014). Iodinated contrast media cause direct tubular cell damage, leading to oxidative stress, low nitric oxide, and impairment of tubuloglomerular feedback. Am J Physiol Renal Physiol.

[15] Arab HH, Salama SA, Maghrabi IA (2018). Camel milk attenuates methotrexate-induced kidney injury via activation of PI3K/Akt/eNOS signaling and intervention with oxidative aberrations. Food Funct.

[16] Liu T, Fang Y, Liu S (2015). Limb ischemic preconditioning protects against contrast-induced acute kidney injury in rats via phosphorylation of GSK-3beta. Free Radic Biol Med.

[17] Kinaci MK, Erkasap N, Kucuk A (2012). Effects of quercetin on apoptosis, NF-kappaB and NOS gene expression in renal ischemia/reperfusion injury. Exp Ther Med.

[18] Havasi A, Borkan SC (2011). Apoptosis and acute kidney injury. Kidney Int.

[19] Liu TQ, Luo WL, Tan X (2014). A novel contrast-induced acute kidney injury model based on the 5/6-nephrectomy rat and nephrotoxicological evaluation of iohexol and iodixanol in vivo. Oxid Med Cell Longev.

[20] Heyman SN, Goldfarb M, Shina A (2003). *N*-Acetylcysteine ameliorates renal microcirculation: studies in rats. Kidney Int.

[21] Heyman SN, Goldfarb M, Carmeli F (1998). Effect of radiocontrast agents on intrarenal nitric oxide (NO) and NO synthase activity. Exp Nephrol.

[22] Raleigh JA, Koch CJ (1990). Importance of thiols in the reductive binding of 2-nitroimidazoles to macromolecules. Biochem Pharmacol.

[23] Nygren A, Ulfendahl HR, Hansell P (1988). Effects of intravenous contrast media on cortical and medullary blood flow in the rat kidney. Invest Radiol.

[24] Liss P, Nygren A, Olsson U (1996). Effects of contrast media and mannitol on renal medullary blood flow and red cell aggregation in the rat kidney. Kidney Int.

[25] Beltowski J (2010). Hypoxia in the renal medulla: implications for hydrogen sulfide signaling. J Pharmacol Exp Ther.

[26] Liss P (1997). Effects of contrast media on renal microcirculation and oxygen tension. An experimental study in the rat. Acta Radiol Suppl.

[27] Liss P, Nygren A, Erikson U (1998). Injection of low and iso-osmolar contrast medium decreases oxygen tension in the renal medulla. Kidney Int.

[28] Siedek F, Persigehl T, Mueller RU (2018). Assessing renal changes after remote ischemic preconditioning (RIPC) of the upper extremity using BOLD imaging at 3T. MAGMA.

[29] Robert R, Vinet M, Jamet A (2017). Effect of non-invasive remote ischemic preconditioning on intra-renal perfusion in volunteers. J Nephrol.

[30] Szijarto A, Hahn O, Lotz G (2006). Effect of ischemic preconditioning on rat liver microcirculation monitored with laser Doppler flowmetry. J Surg Res.

[31] Koti RS, Yang W, Dashwood MR (2002). Effect of ischemic preconditioning on hepatic microcirculation and function in a rat model of ischemia reperfusion injury. Liver Transpl.

[32] Zvara D, Zboyovski JM, Deal DD (2004). Spinal cord blood flow after ischemic preconditioning in a rat model of spinal cord ischemia. Sci World J.

[33] Agmon Y, Peleg H, Greenfeld Z (1994). Nitric oxide and prostanoids protect the renal outer medulla from radiocontrast toxicity in the rat. J Clin Invest.

[34] Rassaf T, Totzeck M, Hendgen-Cotta UB (2014). Circulating nitrite contributes to cardioprotection by remote ischemic preconditioning. Circ Res.

[35] Peng B, Guo QL, He ZJ (2012). Remote ischemic postconditioning protects the brain from global cerebral ischemia/reperfusion injury by up-regulating endothelial nitric oxide synthase through the PI3K/Akt pathway. Brain Res.

[36] Gao X, Zhang H, Takahashi T (2008). The Akt signaling pathway contributes to postconditioning's protection against stroke; the protection is associated with the MAPK and PKC pathways. J Neurochem.

[37] Hausenloy DJ, Tsang A, Yellon DM (2005). The reperfusion injury salvage kinase pathway: a common target for both ischemic preconditioning and postconditioning. Trends Cardiovasc Med.

[38] Fulton D, Gratton JP, McCabe TJ (1999). Regulation of endothelium-derived nitric oxide production by the protein kinase Akt. Nature.

[39] Hashiguchi A, Yano S, Morioka M (2004). Up-regulation of endothelial nitric oxide synthase via phosphatidylinositol 3-kinase pathway contributes to ischemic tolerance in the CA1 subfield of gerbil hippocampus. J Cereb Blood Flow Metab.

[40] Zhu J, Chen X, Wang H (2015). Catalpol protects mice against renal ischemia/reperfusion injury via suppressing PI3K/Akt-eNOS signaling and inflammation. Int J Clin Exp Med.

[41] Li XD, Cheng YT, Yang YJ (2012). PKA-mediated eNOS phosphorylation in the protection of ischemic preconditioning against no-reflow. Microvasc Res.

[42] Yang C, Talukder MA, Varadharaj S (2013). Early ischaemic preconditioning requires Akt- and PKA-mediated activation of eNOS via serine1176 phosphorylation. Cardiovasc Res.

[43] Zhang S, Xin H, Li Y (2013). Skimmin, a coumarin from hydrangea paniculata, slows down the progression of membranous glomerulonephritis by anti-inflammatory effects and inhibiting immune complex deposition. Evid Based Complement Alternat Med.

[44] Sen Z, Jie M, Jingzhi Y (2017). Total coumarins from *Hydrangea paniculata* protect against cisplatin-induced acute kidney damage in mice by suppressing renal inflammation and apoptosis. Evid Based Complement Alternat Med.

[45] Sendeski MM (2011). Pathophysiology of renal tissue damage by iodinated contrast media. Clin Exp Pharmacol Physiol.

[46] Wink DA, Miranda KM, Espey MG (2001). Mechanisms of the antioxidant effects of nitric oxide. Antioxid Redox Signal.

[47] Koivisto A, Pittner J, Froelich M (1999). Oxygen-dependent inhibition of respiration in isolated renal tubules by nitric oxide. Kidney Int.

[48] Koivisto A, Matthias A, Bronnikov G (1997). Kinetics of the inhibition of mitochondrial respiration by NO. FEBS Lett.

[49] Liss P, Hansell P, Fasching A (2016). Iodinated contrast media inhibit oxygen consumption in freshly isolated proximal tubular cells from elderly humans and diabetic rats: Influence of nitric oxide. Ups J Med Sci.

[50] Ii M, Nishimura H, Iwakura A (2005). Endothelial progenitor cells are rapidly recruited to myocardium and mediate protective effect of ischemic preconditioning via "imported" nitric oxide synthase activity. Circulation.

[51] Liu H, Wu R, Jia RP (2013). Ischemic preconditioning increases endothelial progenitor cell number to attenuate partial nephrectomy-induced ischemia/reperfusion injury. PLoS ONE.

